# EXPLORING FIVE WISHES AND END-OF-LIFE CARE PLANNING IN YOUNG ADULTS

**DOI:** 10.1093/geroni/igac059.2518

**Published:** 2022-12-20

**Authors:** Taylor Perre, Arianna Stone, Yuchi Young

**Affiliations:** Home Care Association of New York State, Albany, New York, United States; Home Care Association of New York State, Albany, New York, United States; SUNY at Albany School of Public Health, Rensselaer, New York, United States

## Abstract

Introduction. Advance care planning (ACP) allows individuals to plan ahead and express their preferences for medical treatment and care options to health care providers, family, and loved ones before they are no longer able to make or voice decisions due to the event of a serious illness or injury. Advance directives (ADs) allow individuals to record their preferences. While unintentional injuries are the leading cause of death among young adults, limited studies focus on ACP, ADs, and end-of-life treatment and care. Our study aims to (1) examine the perspectives of young adults towards Five Wishes, and (2) measure their preferences related to personal, emotional, spiritual, and medical values in end-of-life care planning. Methods. Data were collected using Five Wishes and a one-time questionnaire. Participants include graduate students (n=30) at a New York State university. The average age was 24 years old (60% were female, 60% White, and 27% Black). Results. In the case of permanent and severe brain damage without expectation to wake up or recover, 63% do not want life-support treatment. In the event of coma without expectation to wake up or recover, 53% do not want life-support treatment. When close to death, 80% want to have religious or spiritual readings and well-loved - poems read aloud. Conclusions. Young adults are capable of making their decisions regarding appointing a health care proxy and giving specific instructions for personal, emotional, spiritual, and medical care. The present findings intend to make contributions in promoting population-based healthcare decision-making, education, and awareness.

